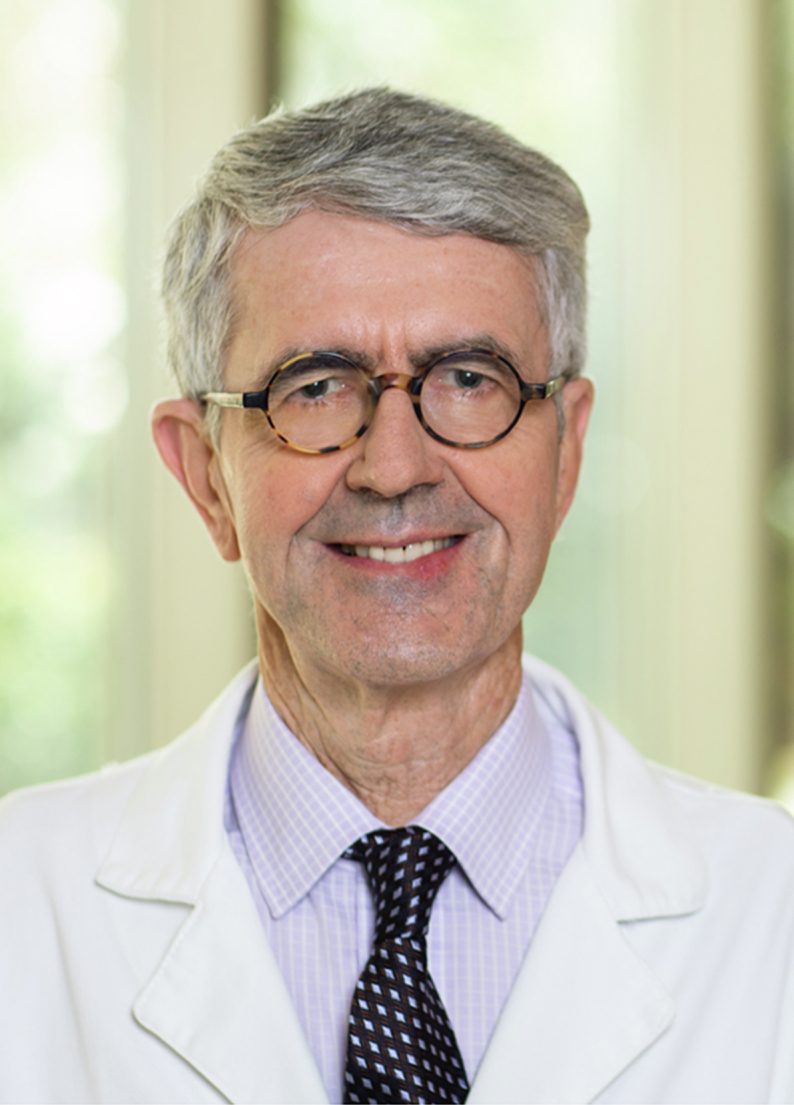# Isolated Hepatitis B Core Antibody

**DOI:** 10.1016/j.gastha.2023.03.012

**Published:** 2023-03-15

**Authors:** Paul Martin, Lawrence S. Friedman

**Affiliations:** 1 Miller School of Medicine, University of Miami, Miami, Florida; 2 Newton-Wellesley Hospital, Newton, MA; Massachusetts General Hospital, Harvard Medical School, and Tufts University School of Medicine, Boston, Massachusetts

The most durable marker of prior hepatitis B virus (HBV) infection after clearance of hepatitis B surface antigen (HBsAg) is immunoglobulin (Ig) G core antibody (anti-HBc), because antibody to hepatitis B surface antigen (anti-HBs) often declines over time. Detection of an “isolated” anti-HBc, without other HBV serologic markers, suggests one of three possibilities: 1) remote resolved HBV infection with immunity; 2) continuing infection with low-level viral replication, or 3) a false-positive anti-HBc result. Administration of a single dose of HBV vaccine should prompt an anamnestic response with production of anti-HBs if the presence of IgG anti-HBc is due to remote resolved HBV infection. Concomitant viral infection with hepatitis C virus or HIV also suggests that the anti-HBc result is a true positive. Isolated IgG anti-HBc without anti-HBs due to continued low-level HBV replication may be confirmed by detection of HBV DNA in serum. Alternatively, the anti-HBc may be a false-positive result. Implications of a true-positive isolated IgG anti-HBc result include the possibilities of transmission of HBV infection to others through blood or organ donation and of HBV reactivation during chemotherapy or immune modulating therapy. HBV reactivation can lead to hepatic decompensation. This outcome can be prevented by initiating antiviral therapy before beginning chemotherapy or immunotherapy.

**Figure:**
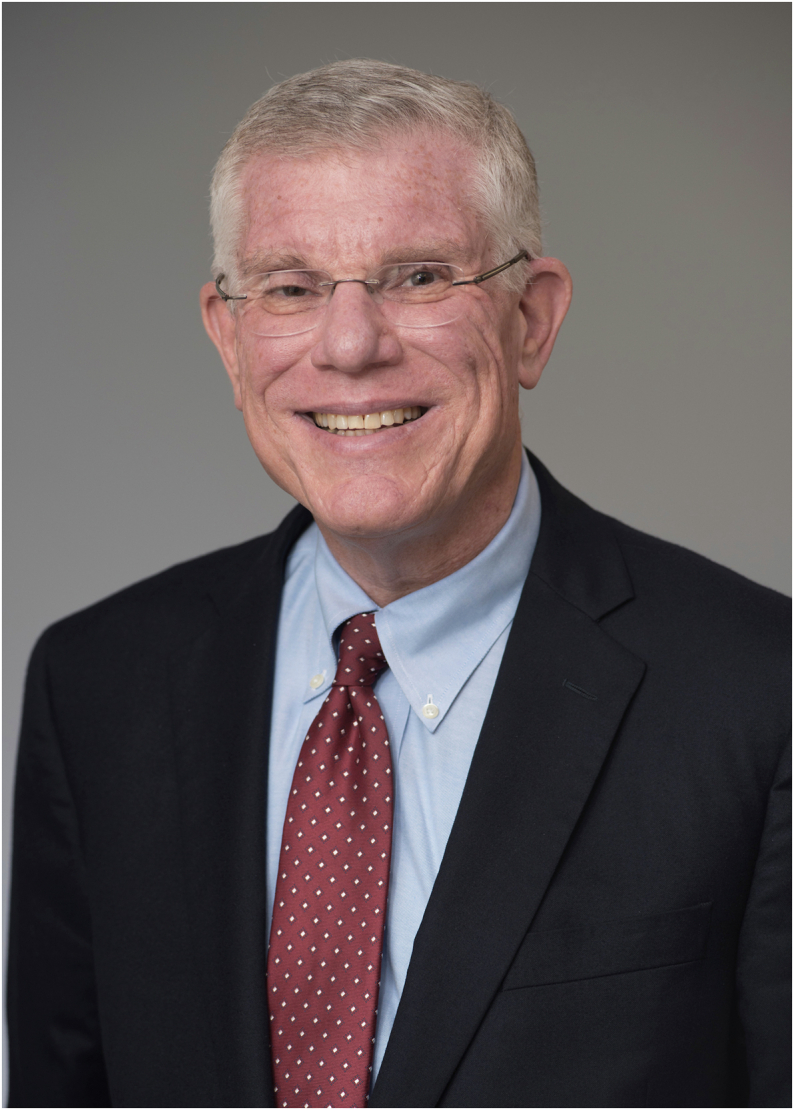

**Figure:**